# Multimedia Bootcamp: a health sciences library provides basic training to promote faculty technology integration

**DOI:** 10.1186/1742-5581-3-3

**Published:** 2006-04-25

**Authors:** Ellen C Ramsey

**Affiliations:** 1 Claude Moore Health Sciences Library, University of Virginia, Charlottesville, VA 22908, USA

## Abstract

**Background:**

Recent research has shown a backlash against the enthusiastic promotion of technological solutions as replacements for traditional educational content delivery. Many institutions, including the University of Virginia, have committed staff and resources to supporting state-of-the-art, showpiece educational technology projects. However, the Claude Moore Health Sciences Library has taken the approach of helping Health Sciences faculty be more comfortable using technology in incremental ways for instruction and research presentations. In July 2004, to raise awareness of self-service multimedia resources for instructional and professional development needs, the Library conducted a "Multimedia Bootcamp" for nine Health Sciences faculty and fellows.

**Methods:**

Case study.

**Results:**

Program stewardship by a single Library faculty member contributed to the delivery of an integrated learning experience. The amount of time required to attend the sessions and complete homework was the maximum fellows had to devote to such pursuits. The benefit of introducing technology unfamiliar to most fellows allowed program instructors to start everyone at the same baseline while not appearing to pass judgment on the technology literacy skills of faculty. The combination of wrapping the program in the trappings of a fellowship and selecting fellows who could commit to a majority of scheduled sessions yielded strong commitment from participants as evidenced by high attendance and a 100% rate of assignment completion. Response rates to follow-up evaluation requests, as well as continued use of Media Studio resources and Library expertise for projects begun or conceived during Bootcamp, bode well for the long-term success of this program.

**Conclusion:**

An incremental approach to integrating technology with current practices in instruction and presentation provided a supportive yet energizing environment for Health Sciences faculty. Keys to this program were its faculty focus, traditional hands-on instruction, unrestricted access to technology tools and support, and inclusion of criteria for evaluating when multimedia can augment pedagogical aims.

## Background

Recent research has shown a backlash against the enthusiastic promotion of technological solutions as replacements for traditional educational content delivery[1,2]. Many institutions, including the University of Virginia, have committed staff and resources to supporting state-of-the-art, showpiece educational technology projects. However, the Claude Moore Health Sciences Library has taken the approach of helping Health Sciences faculty be more comfortable using technology in incremental ways for their instruction and research presentations. As a natural outgrowth of our support of common technologies such as image scanning, PDF creation, and electronic slide presentations, in 2002 the Library created and funded a "Media Studio" which includes the common technologies plus more advanced multimedia applications and hardware. While use of the Media Studio has steadily increased over the past two years, the addition in 2004 of a faculty member for educational technology has allowed the Library to reach out to faculty beyond the early adopters who have already found ways to integrate multimedia into their academic endeavors. In July 2004, to raise awareness of the Media Studio and self-service multimedia resources for instructional and professional development needs, the Library conducted a "Multimedia Bootcamp" for nine Health Sciences faculty and fellows.

## Methods

### Staffing and support

Support for the program came from a collaboration of Library faculty and local and national vendors. Program design, administration, and instruction were coordinated by the Library's Educational Technology Coordinator. This Library faculty member served as the program's director and lead instructor, and recruited other Library staff and faculty to teach appropriate program topics. The approach of the program was to provide intensive, hands-on practice in creating multimedia content. In support of this approach, each instructional session was staffed by two or three staff dedicated to providing individualized assistance, in addition to the instructor. Participants were also given assignments to complete between sessions. Support from the Apple Seeds for Education Program gave each program fellow exclusive use of a multimedia capable laptop and video camera for the duration of the month-long program. Digital still cameras, recordable media, and a large-format poster to used an example of the kind of print media possible with digital imaging technology were all donated by vendors. See Table 1 for a summary of equipment and supplies. In acknowledgement of their support, each vendor was invited to participate in a Multimedia Vendor Day open to the University community and interested members of the public.

**Table 1:** Summary of Multimedia Bootcamp 2004 Equipment and Supplies

**Item**	**Contributed by**	**Value**
Loan of 10 PowerBook laptops, 10 digital video cameras	Apple Computer, Inc.	$28,480
Loan of 12 digital still cameras	Crutchfield Corporation	$6,000
Donation of poster printing	International Imaging Inc.	$175
Donation of consumable digital media (miniDV tape, CD-R, DVD-R)	Cavalier Computers	$200
Incidental expenses (catering, office supplies, printing, etc)	Claude Moore Health Sciences Library (host)	$700
Total value of equipment loans and in-kind contributions		$35,555

### Nomination and selection process

The Multimedia Bootcamp was designed as a fellowship program to secure the commitment of faculty participants. Rather than sign up for a selection of training classes or consultations with knowledgeable Library staff, faculty were encouraged to nominate themselves or colleagues as "Bootcamp Fellows" willing to participate in a four-week program. Nominees were asked to submit a short statement of how they planned to apply skills from the Bootcamp curriculum to benefit their academic endeavors, and were asked to detail any conflicts with the scheduled sessions. Preference was given to participants who were able to attend a majority of the sessions. The selection committee consisted of four Library faculty members familiar with the capabilities of the multimedia studio or faculty technology needs. Faculty selected for the program received letters informing them of their nomination as fellows and detailing their responsibilities for safekeeping of loaned equipment. Such efforts may seem inconsequential to providing instruction, but it was our hope that wrapping the program in the trappings of a fellowship would increase the level of interest and commitment from busy clinical and teaching faculty for a program which required a significant commitment of time (two to four hours per week for four weeks).

### Equipment and teaching space

Five of the six sessions were held in the Library's Tolleson Wireless Classroom, which has moveable tables and chairs, wireless network connectivity, a reliable projection system, and plenty of electrical outlets for cameras, computers, and presentation equipment. Given the right environment, it was expected that relaxed but focused learning could take place.

Fellows had unrestricted access to high-quality multimedia equipment for the duration of the program. The ten multimedia equipped PowerBook G4 laptops and ten consumer grade digital video cameras made it possible for each fellow to have a dedicated set of equipment to use both in class and for homework assignments. The still digital cameras were assigned to fellows for the first two weeks of the program, including the period between the first two sessions.

### Instructional content and delivery

A series of six two-hour sessions spread over four weeks in July focused on skills integrating multimedia into instructional and presentation materials. Footage of one instructional session was captured [see Additional file Video 1]. The schedule for the program content is detailed in Table 2.

**Table 2:** Multimedia Bootcamp 2004 Session Schedule

**Date**	**Content**
Thursday, July 8	Effective multimedia use; working with digital still cameras and images
Thursday, July 15	Digital video capture; introduction to digital video editing
Wednesday, July 21	Multimedia Vendor Day
Thursday, July 22	Digital video editing continued
Wednesday, July 28	Sharing digital content through conference posters
Thursday, July 29	Sharing digital content electronically

All sessions were two hours in duration, and were held in the Claude Moore Health Sciences Library's Learning Resources Center.

While most of the skills taught during the program were within the normal repertoire of Library classes and consultations, content was created with the needs and interests of Health Sciences faculty in mind. An instructional design perspective on the effective use of multimedia was introduced during the first session to reinforce the pedagogical finding that merely adding electronic educational resources does not necessary improve instruction [3,4]. Unlike stand-alone classes on one aspect of multimedia (for example, the Library's popular image scanning and enhancement class), the extended program format lent itself to using the same example project scenario through all five hands-on sessions. With the help of a resident who had used the Library's Media Studio for several projects in the past, we were able to construct a neurology case study. By adhering to the University's patient confidentiality and privacy regulations, we were able to obtain still and video images of a patient with ataxia. Additional resources on stroke and ataxia were added to the sample content from the Library's image and monograph collections. These relevant resources formed the basis for both an example of educational DVD, which could be employed to teach neurological assessment techniques, and the footage used to teach video capture and editing techniques.

While in-class demonstrations and hands-on exercises used the neurology case study materials, fellows were encouraged to use their own content for the weekly homework assignments. Compliance with completing assignments was 100%. Table 3 details the homework assignments related to each session.

**Table 3:** Multimedia Bootcamp 2004 Homework Assignments

**Date**	**Assignment**
Thursday, July 8	• Using the digital still camera, capture at least five images of two different subjects • Using Media Studio scanners, capture one scanned image (optional)
Thursday, July 15	• Using the digital video camera, film at least two scenes for a total of at least 15 minutes of video • Import captured footage onto PowerBook using video camera and iMovie
Thursday, July 22	• Completed video editing to prepare for electronic sharing via DVD, PowerPoint, or Web

### Publicity

The program was promoted with the endorsement of faculty who had used the Library's Media Studio services in the past, as well as through various Health Sciences faculty outreach lists and publications. In addition to direct e-mail to Media Studio patrons asking for nominations of appropriate colleagues, e-mail solicitations for nominations were sent to lists of nursing, clinical, and basic sciences faculty. Notices were also published in the Library's monthly newsletter, the School of Nursing's weekly faculty communiqué, the hospital's faculty and staff newsletter, through the Library Departmental Liaison Program, and contacts with the Housestaff Office, which is responsible for teaching fellowship programs.

## Results

### Staffing and support

The leadership of a single Library faculty member to shepherd the inaugural program to completion required approximately 50% of the person's time during the two months leading up to the program. While it was a significant staffing commitment, the time devoted contributed to the delivery of an integrated learning experience. The expectation that fellows would ply us with many questions and requests for individualized support between sessions turned out to be false. The month of the program was actually rather quiet except for the sessions themselves, leading us to conclude that the amount of time required to attend the sessions and complete homework assignments was the maximum fellows had to devote to such pursuits.

The presence at each session of two or three additional staff devoted to individual hands-on support was effective to ensure that fellows had adequate support for complex tasks such as image manipulation and video editing techniques. However, except for the most advanced session when fellows were completing the final edits on their video projects, one hands-on assistant would probably have been sufficient. While most fellows came into the program with little experience creating multimedia, they seemed more comfortable experimenting with the technology than we anticipated when designing the program. The inclusion at the outset of the program of an overview of the Macintosh environment also served to bring all of the fellows onto a level playing field. It can be difficult to imparting basic technology skills to faculty without being perceived as patronizing of their computer literacy[5]. The benefit of using the Macintosh platform, which was unfamiliar to all but two of the fellows, meant we could introduce basic concepts like file management and mouse/touchpad techniques in the context of learning what was different from the Windows platform, thus starting everyone at the same baseline while not appearing to pass judgment on faculty in this area.

The Multimedia Vendor Day fulfilled its promise to offer sponsoring vendors access to prospects in return for their support of the Bootcamp. The four sponsors put together excellent displays and presentations, and hosted over 70 visitors from the health sciences center, University, and community, footage of this event was captured [see Additional file Video 2]. All of the vendors expressed their desire to sponsor the Bootcamp in the future, as they felt it provided them an unusual opportunity to showcase their support for the integration of multimedia technology in the health sciences and higher education environment.

### Nomination and selection process

Our initial expectations of interest in the program by potential fellows were far exceeded. The original plan was for a maximum of eight Health Sciences faculty to receive fellowships. To reflect the Library's patron base, we hoped to offer fellowships to at least one basic sciences researcher, one member of the School of Nursing faculty, and the remainder to clinical faculty or teaching fellows. Because we received credible nominations from thirteen faculty members or teaching fellows, the program was expanded to support nine Bootcamp fellows and a waiting list was created for those applicants for whom we did not have space in the inaugural program. While we would have liked to admit all thirteen applicants, we were limited to ten laptops and cameras, and one set was needed for the instructor and hands-on instruction. In addition, it was important to keep the group to a relatively small size to facilitate individualized, hands-on assistance during instructional sessions. With nine fellows, one lead instructor, and two hands-on assistants, we were able to keep the ratio of instructional staff to fellows at three to one.

Selection of fellows followed the guidelines outlined in the methods section. Nominations were collected and evaluated in order of submission, which was easy to track, given the required e-mail format of submissions. Preference was given to nominees who were able to commit to a majority of scheduled sessions. Since all nominees included a clear statement of their academic goals for the program, it was not necessary for us to eliminate any application based on that criterion. Instead, we were able to rank nominations by arrival date, availability for sessions, and representativeness of the Library's patron base. Selected goal statements included:

A professor and pediatric urologist said, "I...perform quite a bit of reconstructive surgery on infants and children. Our department has acquired a Da Vinci Robot Assisted Surgical System, and I have already completed my first case using this exciting minimally invasive surgical technology (the first pediatric case at UVA). There are relatively few pediatric institutions in the country with this technology, and I will [be] presenting data at regional and national talks in the near future. The surgeries are completely recorded in digital video, and I would very much like to learn how to edit, integrate, and share this important information with my pediatric surgical sub-specialists."

An emergency medicine professor who provides paramedic education said, "I envision utilizing the skills learned to refine and invigorate offerings of [the local] paramedic program didactics, where we are trying to increasingly shift to online offerings through Blackboard and web-enhanced curricula. Now that we have obtained national accreditation, my next project is to begin pursuing national accreditation to provide online continuing education programs for EMS providers. I have [PC-based video editing equipment] and have imported and used some small clips embedded in PowerPoint lectures given at regional and statewide continuing education lectures. I would like to learn how to do it with greater skill and finesse, [since] so far my knowledge in this area is all self-taught."

A veterinarian who is a lead animal researcher said, "I am responsible for training all individuals using laboratory animals for research purposes at the University of Virginia. This training currently includes a monthly orientation seminar, a monthly hands-on rodent handling workshop, maintaining a website with training information, and individualized training as needed. The orientation seminar format is [PowerPoint] presentations which could be improved upon by better use of multimedia. I am also writing a chapter on rodent surgery for an on-line textbook which allows use of digital still-images and video. Finally, I plan to expand the training services offered to include a series of interactive web-based training modules. One of these will be an annual re-training mandatory for all animal researchers; the others will be specific to a particular species or procedure. I believe that a class in multimedia skills would be an immense asset in helping me to develop new teaching and presentation tools that are interesting, relevant, and effective."

A professor of graduate nursing said "I am currently teaching an entirely on line graduate level class in the UVa School of Nursing using Blackboard as the platform and I would like to enhance my skills in media use beyond the use of [PowerPoint]. I am also involved in continuing education in geriatrics through the UVa Health System and would like to explore how the geriatric content NetLearning [the Health System's online education portal for staff] educational modules could be more appealing and interactive through the use of media."

The entire cohort included an associate professor in cardiovascular medicine, assistant professors in obstetrics and gynecology, physical medicine/rehabiliation, emergency medicine, nursing, pediatric urology; and two fellows in family medicine.

The combination of wrapping the program in the trappings of a fellowship and selecting fellows who could commit to a majority of scheduled sessions meant that not only were attendance levels extremely high (all but one fellow attended or made up all of the required sessions), but participation in homework assignments was also 100%. Fellows were invested enough in the program that they requested certificates of completion at the conclusion of the program. The program administrator had thought this type of acknowledgement would be unimportant to this highly credentialed group, so had not prepared certificates for distribution at the last session. In response to the fellows' request, certificates and copies of the class portrait were prepared and mailed out following the final session, along with follow-up evaluation materials. Follow-up evaluation comments, as well as continued use of Media Studio resources and Library expertise for projects begun or conceived of during Bootcamp, bode well for the long-term success of this program.

### Equipment and teaching space

Five of the six sessions were held in the Library's Tolleson Wireless Classroom. This space proved ideal for the intimate, hands-on nature of the instruction. Moveable tables and chairs, wireless network connectivity, a reliable projection system, and plenty of electrical outlets for cameras, computers, and presentation equipment provided the right environment for relaxed but focused learning. Fellows and instructional staff alike were able to move about the room for help and collaboration during sessions. The remaining session, which required the use of Windows workstations for integrating multimedia into the ubiquitous PowerPoint for Windows, was conducted in the Library's traditional computer classroom. The different dynamic of that session (faces hidden behind stationery computer monitors and little room for fellows to work with their laptops) highlighted the benefits of the more flexible setting used for the other sessions. The wireless classroom also served as a viable setting for the Multimedia Vendor Day event, since the numerous network connections and moveable furniture allowed the teaching space to be configured for multiple vendor displays and effective crowd flow. The attached video shows the wireless classroom during the second hands-on session and also during the vendor day.

Fellows' unrestricted access to high-quality multimedia equipment for the duration of the program gave them the ability to take the equipment out of the Library and into labs, classrooms, and their diverse educational settings. This flexibility meant that fellows were able to immediately apply what was learned in class to their real world instructional circumstances.

### Evaluation

A Kirkpatrick Level One Evaluation was collected at the conclusion of the final session. Eight of the nine fellows responded to the level one survey. The questions and their results are shown in Table 4.

**Table 4:** Kirkpatrick level one evaluation, conducted July 29, 2004

**Please rate the following statements on a scale of 1 (strongly disagree) to 5 (strongly agree):**	**Average Score (out of 5)**
I learned concepts and skills I can put into practice.	4.875
The way the instruction was organized helped me learn.	4.75
"Homework" assignments were relevant and useful.	5
Hands-on exercises were relevant and useful.	4.875
Tools (software/hardware) were appropriate.	4.875
I would recommend this program to others interested in integrating multimedia into presentation and instruction.	4.75
I plan to use the Media Studio for multimedia projects in the future.	4.875
	
**Please rate the overall quality of the following program components on a scale of 1 (poor) to 5 (excellent):**	**Average Score (out of 5)**
Program Contents	4.75
Printed materials/handbook	4.75
Instructors' knowledge/preparation	5
Equipment	5
	
**The most helpful parts of this program were: (selected comments)**	
• The actual hands on part of the class	
• Learning about how to put photos into PowerPoint	
• Learning how to add an MPEG into PowerPoint	
• The instructors were helpful	
• Intro to equipment/software/services in Media Center and online (imagesMD)	
	
**The least helpful parts of this program were: (selected comments)**	
• The iMovie part was less helpful	
• Using iPhoto – most users have PCs	
• Having to carry heavy equipment to and fro (but I cannot see how to avoid it)	

A debriefing meeting including available Library staff that participated in Bootcamp as instructors, hands-on assistants, or logistical help, was held six weeks after the program's conclusion. By combining a review of participant feedback with the staff debriefing, we will implement changes small and large in future programs. Key revisions are anticipated to include:

• Adding an intensive, two-day session specifically for staff that support faculty presentation and instruction projects

• Moving the PowerPoint integration topic to the final session to allow more time for experimentation with DVD burning between sessions five and six

• Dropping digital still camera use in favor of more time spent editing still images

• Inclusion of previous Bootcamp projects as examples

• Merging of the Multimedia Vendor Day with the Library's InfoFair, a popular yearly information and technology event, which drew over 700 visitors in 2003

A Kirkpatrick Level Three Evaluation was conducted eight weeks after the program's conclusion. Surveys were mailed to fellows with the following three questions:

1. Have you used any of the concepts that you learned in Multimedia Bootcamp? If yes, briefly describe how.

2. If you haven't yet used any of the concepts or skills that you learned in Bootcamp, do you anticipate you will? Why, or why not?

3. What can we do to help you get the most out of the resources you learned about?

Five of the nine fellows responded to the survey. Three respondents indicated they had already used skills acquired at Bootcamp, including integrating multimedia with PowerPoint, creating posters with digital images, purchasing and using a new digital camera, and making a prototype teaching video. The respondents who had not yet applied any of the new skills indicated that they anticipated using them when writing new lectures or needing to integrate video clips from surgical procedures into PowerPoint. Responses to the question about how the Library could help fellows utilize our resources included requests for assistance with planned projects, anticipation of using Media Studio resources, and appreciation for assistance already provided.

In addition to the level three survey responses, we have been able to see the longer-term effects of the program through requests to Library staff for assistance on projects originating at Bootcamp. In particular:

The emergency medicine professor who provides paramedic education worked on a prototype DVD for a trauma-response training program for emergency medicine first-responders. Since the conclusion of bootcamp, she has worked the national organization that sponsors this training on specifications for the final DVD, which will replace an out-of-date VHS resource currently used for this certification training. Recently, we have collaborated with this clinician on shooting the video for this project so that we may use it as the basis for sample exercises during future Bootcamp programs.

The veterinarian and lead animal researcher shot footage of the animal lab facility, then encoded it for inclusion in PowerPoint presentations highlighting the requirements for training in humane care and use of animals at the University of Virginia. The addition of live video to these presentations will be helpful to her role of conveying key concepts regarding use of animals in biomedical research.

## Conclusion

An incremental approach to integrating technology with current practices in instruction and presentation provided a supportive yet energizing environment for busy Health Sciences faculty. Keys to this program were its faculty focus, traditional hands-on instruction, unrestricted access to technology tools and support, and inclusion of criteria for evaluating when multimedia can augment pedagogical aims.

For libraries interested in presenting a faculty multimedia training program, lessons which can be learned from our experience include:

• Design and promote the program as a fellowship or other prestigious series

• Enlist the support of multimedia services vendors

• Espouse criteria for appropriate use of multimedia in instruction and presentation

• Enlist familiar instructional strategies to gently introduce new technologies and approaches to faculty wary of the "bleeding edge."

## Abbreviations

PDF: Portable Document Format; a standard developed by Adobe^®^, which makes text files readable on different operating systems

CD-R: Compact Disc (recordable); optical storage which holds at least 700 Megabytes of data

DVD-R: Digital Video Disc (recordable); optical storage which holds at least 4.7 Gigabytes of data, enough for a full-length movie.

VHS: Video Home System; the common 1/2 inch consumer videotape format

## Competing interests

The author(s) declare that they have no competing interests.

## Authors' contributions

ER designed and administered the Bootcamp program and drafted the manuscript.

## Supplementary Material

Additional File 1 Video 1 – Multimedia Bootcamp 2004 Instructional Session. QuickTime version: MMB_video1.mov. Windows Media Player version: MMB_video1.wmv. Click here for file

Additional File 2 Video 2 – Multimedia Bootcamp 2004 Vendor Day. QuickTime version: MMB_video2.mov. Windows Media Player version: MMB_video2.wmv. Click here for file
